# Regulation of Ghrelin Receptor by Periodontal Bacteria *In Vitro* and *In Vivo*

**DOI:** 10.1155/2017/4916971

**Published:** 2017-11-29

**Authors:** Marjan Nokhbehsaim, Anna Damanaki, Andressa Vilas Boas Nogueira, Sigrun Eick, Svenja Memmert, Xiaoyan Zhou, Shanika Nanayakkara, Werner Götz, Joni Augusto Cirelli, Andreas Jäger, James Deschner

**Affiliations:** ^1^Section of Experimental Dento-Maxillo-Facial Medicine, Center of Dento-Maxillo-Facial Medicine, University of Bonn, Bonn, Germany; ^2^Department of Diagnosis and Surgery, School of Dentistry at Araraquara, São Paulo State University (UNESP), Araraquara, SP, Brazil; ^3^Department of Periodontology, Laboratory of Oral Microbiology, University of Bern, Bern, Switzerland; ^4^Department of Orthodontics, Center of Dento-Maxillo-Facial Medicine, University of Bonn, Bonn, Germany; ^5^Faculty of Dentistry, University of Sydney, Sydney, Australia; ^6^Institute of Dental Research, Westmead Centre for Oral Health and Westmead Institute for Medical Research, Sydney, Australia; ^7^Noel Martin Visiting Chair, Faculty of Dentistry, University of Sydney, Sydney, Australia

## Abstract

Ghrelin plays a major role in obesity-related diseases which have been shown to be associated with periodontitis. This study sought to analyze the expression of the functional receptor for ghrelin (GHS-R1a) in periodontal cells and tissues under microbial conditions *in vitro* and *in vivo*. The GHS-R1a expression in human periodontal cells challenged with the periodontopathogen *Fusobacterium nucleatum*, in gingival biopsies from periodontally healthy and diseased individuals, and from rats with and without ligature-induced periodontitis was analyzed by real-time PCR, immunocytochemistry, and immunofluorescence. *F. nucleatum* induced an initial upregulation and subsequent downregulation of GHS-R1a in periodontal cells. In rat experimental periodontitis, the GHS-R1a expression at periodontitis sites was increased during the early stage of periodontitis, but significantly reduced afterwards, when compared with healthy sites. In human gingival biopsies, periodontally diseased sites showed a significantly lower GHS-R1a expression than the healthy sites. The expression of the functional ghrelin receptor in periodontal cells and tissues is modulated by periodontal bacteria. Due to the downregulation of the functional ghrelin receptor by long-term exposure to periodontal bacteria, the anti-inflammatory actions of ghrelin may be diminished in chronic periodontal infections, which could lead to an enhanced periodontal inflammation and tissue destruction.

## 1. Introduction

Periodontitis represents one of the most prevalent diseases affecting systemic health and the quality of life [1, 2]. It is a multifactorial inflammatory disease elicited by a complex of several bacterial species in the subgingival biofilm, such as *Porphyromonas gingivalis*, *Tannerella forsythia*, *Treponema denticola*, *Aggregatibacter actinomycetemcomitans*, and *Fusobacterium nucleatum*. Smoking, genetic predisposition, mental stress, and several systemic diseases are also important contributing factors to the initiation and progression of periodontitis [3]. The bacterial species interact with infiltrating and resident host cells, thereby causing the release of a broad array of inflammatory mediators and proteases, such as interleukin- (IL-) 1*β*, IL-6, IL-8, tumor necrosis factor-*α* (TNF), cyclooxygenase-2 (COX2), chemokine CC motif ligand 2 (CCL2), and matrix metalloproteinases (MMPs) [4, 5]. If the immunoinflammatory response is exaggerated and/or prolonged, irreversible destruction of periodontal tissues including periodontal ligament (PDL) and alveolar bone can occur, resulting in periodontal pocket formation and even tooth loss.

A substantial body of studies has shown that periodontitis is associated with the onset, development, and/or progression of systemic diseases, such as cardiovascular diseases, type 2 diabetes, obesity, and metabolic syndrome [6–9]. However, the pathomechanisms underlying these associations are yet to be clarified and require further investigation. It is noteworthy that the role of ghrelin (GHRL) in periodontal health and disease has become the focus of a few recent studies due to its link to obesity as well as its modulatory functions on the immune system [10, 11]. GHRL, which was originally identified as a hormone secreted mainly by gastrointestinal cells, plays a critical role in a range of biological processes, such as regulating food intake, energy balance, body weight as well as sleep, and memory [12–14]. GHRL mediates its actions by binding to its receptor, the growth hormone secretagogue receptor (GHS-R), which has been found in the hypothalamus, pituitary, pancreas, heart, salivary glands, stomach, and many other organs [15, 16]. GHS-R is expressed in two isoforms: type 1a and 1b. As a G protein-coupled receptor, GHS-R1a consists of 366 amino acids with the typical seven transmembrane domains. Upon binding with GHRL, GHS-R1a undergoes a profound change in conformation and triggers a diversity of physiological responses, while the inactive GHS-R variant, the GHS-R1b, does not mediate any effects of GHRL [16]. Until now, only a few studies have focused on the role of GHRL in periodontitis to explore whether GHRL may be involved in the regulation of periodontal inflammatory responses. Briefly, GHRL levels in gingival crevicular fluid (GCF) have been found lower in periodontitis patients when compared with healthy subjects [17]. However, this observation was in contrast to the GHRL levels in serum [18]. GHRL was also detected in saliva as well as in several cells and tissues of the tooth germ, such as inner enamel epithelium, mesenchymal cells, ameloblasts, odontoblasts, and Hertwig's epithelial root sheath [19–21]. Nevertheless, the exact role of the GHRL/GHS-R system in periodontal health and disease is yet to be unraveled. Therefore, the main objective of the present study was to evaluate the expression of GHS-R1a in periodontal cells and tissues under microbial conditions *in vitro* and *in vivo*.

## 2. Materials and Methods

### 2.1. Human PDL Cell Culture and Treatment

This study and the protocols were approved by the Ethics Committee of the University of Bonn, and written informed consent was obtained prior to sample collection (number 043/11). Human PDL cells were isolated from periodontally healthy teeth that were extracted for orthodontic indications. Briefly, the cells were cultured in Dulbecco's minimal essential medium (DMEM, Invitrogen, Karlsruhe, Germany) supplemented with 10% fetal bovine serum (FBS, Invitrogen), 100 U/mL penicillin, and 100 *μ*g/mL streptomycin (Invitrogen) at 37°C in a humidified atmosphere of 5% CO_2_. Cells between the 3rd passage and the 5th passage were seeded (5.0 × 10^4^ cells/well) on culture plates and grown to 80% confluence. One day prior to the experiments, the FBS concentration was reduced to 1%. The medium was changed every second day.

To mimic oral infections by microbial pathogens *in vitro*, oral pathogenic bacteria associated with periodontitis were selected and applied to challenge the PDL cells for up to 2 d: *F. nucleatum* ATCC 25586 (OD_660_: 0.025, 0.05, 0.1, and 0.2), *P. gingivalis* ATCC 33277 (OD_660_: 0.05, 0.1 and 0.2), *A. actinomycetemcomitans Y4* (OD_660_: 0.1), and *T. denticola* (OD_660_: 0.1). Bacteria were suspended in phosphate-buffered saline (PBS) (OD_660_ = 1.0, equivalent to 1.2 × 10^9^ bacterial cells/mL) and subjected twice to ultrasonication (160 W for 15 min). To evaluate the possible anti-inflammatory actions of the GHRL/GHS-R system, PDL cells were preincubated with 20 nM of GHRL (human n-octanoylated ghrelin, Pepta Nova, Sandhausen, Germany) and 10 *μ*g/mL of Toll-like receptor-4 (TLR4) blocking antibody (eBioscience, San Diego, CA, USA), respectively, 45 min prior to the challenge with *F. nucleatum*.

### 2.2. Human Gingival Biopsies

Human gingiva samples were obtained from 10 patients with periodontitis from the Department of Oral Surgery of the University of Bonn during tooth extraction for periodontal indications. Written informed consent and approval of the Ethics Committee of the University of Bonn were obtained (number 043/11). Samples collected from 10 individuals with gingivitis and 10 periodontally healthy donors during wisdom tooth removal or tooth extraction for orthodontic indications were also used [22]. Participants diagnosed with systemic diseases or having smoking habits were excluded. Clinically, gingival index (GI), probing pocket depth (PD), clinical attachment loss (CAL), and radiographic bone loss were assessed. Gingival sites with GI = 0 (no clinical inflammation), PD ≤ 3 mm, no CAL, and no radiographic bone loss were defined as periodontally healthy, and the sites with GI > 1, PD ≥ 5 mm, CAL ≥ 3 mm, and radiographic bone loss were defined as having periodontal disease.

### 2.3. Rat Gingival Biopsies

All animal experimental procedures described in this study were approved by the Ethical Committee on Animal Experimentation at the School of Dentistry at Araraquara, São Paulo State University (UNESP) (protocol number 23/2012), in compliance with the Animal Research: Reporting of *In Vivo* Experiments (ARRIVE) guidelines. A total of 24 male adult Holtzman rats weighing about 300 g were selected and caged in an animal house with provision of standard laboratory food and water ad libitum. Experimental periodontitis in rats was induced by using ligatures, as previously described [23]. Briefly, the animals were randomly divided into two groups. One group was left untreated and served as the control, and the other group was subjected to ligation to induce periodontal disease. A cotton ligature was tied around the cervical area of the maxillary first molars bilaterally. The knot was placed mesially under anesthesia with intramuscular injections of ketamine chlorhydrate 10% (0.08 mL/100 g body weight) and xylazine chlorhydrate 2% (0.04 mL/100 g body weight). At three different time points following ligation (6 d, 8 d, and 12 d), 4 rats from each group were sacrificed and the gingival tissues around the maxillary first molars were carefully dissected for total RNA extraction followed by real-time PCR.

### 2.4. Real-Time Polymerase Chain Reaction

The gene expressions in samples from PDL cells as well as human and rat gingival biopsies were analyzed by quantitative RT-PCR. Total RNA extraction was performed using an RNeasy Mini Kit (Qiagen, Hilden, Germany) according to the manufacturer's protocol. RNA concentration was determined by a NanoDrop ND-2000 (Thermo Fisher Scientific, Wilmington, DE, USA) spectrophotometer, and 500 ng of total RNA was reversely transcribed using the iScript™ Select cDNA Synthesis Kit (Bio-Rad Laboratories, Munich, Germany) at 42°C for 90 min followed by 85°C for 5 min as per the manufacturer's instruction. The analysis of gene expressions of GHS-R1a, CCL2, IL-6, and IL-8 was subsequently performed in triplicate by using QuantiTect Primers (Qiagen), SYBR Green QPCR Master Mix (Bio-Rad), and the iCycler iQ™ Real-Time PCR Detection System (Bio-Rad). Amplification was carried out under the following conditions: initial denaturation at 95°C for 5 min and followed by 40 cycles of denaturation at 95°C for 10 s and combined annealing/extension at 60°C for 30 s. Data were analyzed using the comparative threshold cycle (CT) method with glyceraldehyde-3-phosphate dehydrogenase (GAPDH) as the housekeeping gene.

### 2.5. Immunocytochemistry for GHS-R Detection

PDL cells were grown in the presence or absence of *F. nucleatum* on plastic coverslips (Thermo Fisher Scientific) of 13 mm diameter in 24-well plates for 1 d and 2 d. Cell monolayers were fixed in 4% paraformaldehyde (Sigma-Aldrich, Munich, Germany) at pH 7.4 and room temperature (RT) for 10 min and permeabilized in 0.1% Triton X-100 (Sigma-Aldrich) for 5 min followed by blocking using serum block (Dako, Hamburg, Germany) for 20 min. Afterwards, the cells were labeled with rabbit polyclonal primary antibody to GHS-R (Abcam, Cambridge, UK, 1 : 500) in a humid chamber at 4°C overnight and then incubated with goat anti-rabbit IgG HRP secondary antibody (Dako) for 45 min. The cells were rinsed with PBS (Invitrogen) in between each step. Finally, the cells were mounted with DePeX (SERVA Electrophoresis, Heidelberg, Germany) and the production of GHS-R was assessed with an Axioskop 2 microscope (20×, Carl Zeiss, Germany). The images were captured with an AxioCam MRc camera and analyzed with the AxioVision 4.7 software (Carl Zeiss). Untreated cells were used as a control.

### 2.6. Immunofluorescence for Nuclear Factor-*κ*B p65 Detection

Plastic coverslips (Thermo Fisher Scientific) with growing PDL cells were incubated in the presence or absence of *F. nucleatum* for 90 min. Following the immunocytochemistry method as described above, the cells were fixed and permeabilized and then blocked with nonfat dry milk (Bio-Rad) for 1 h. The slides were subsequently incubated with a rabbit anti-nuclear factor-*κ*B p65 (E498) primary antibody (Cell Signaling Technology, Danvers, MA, USA; 1 : 100) for 90 min at RT. After rinsing with PBS and incubating with CY3-conjugated goat anti-rabbit IgG secondary antibody (Abcam; 1 : 1000) for 45 min at RT, the expression of NF-*κ*B p65 in cells was observed with the ZOE™ Fluorescent Cell Imager (Bio-Rad) with a 20x objective. The images were captured with an integrated digital 5MP CMOS camera. Untreated cells were used as a control.

### 2.7. Immunohistochemistry for GHS-R Detection

Human gingival biopsies from healthy donors and periodontitis patients (*n* = 3) were first fixed in 4% paraformaldehyde (Sigma-Aldrich) for 2 d, dehydrated in an ascending ethanol series (AppliChem, Darmstadt, Germany), and subsequently embedded in paraffin (McCormick Scientific, Richmond, IL, USA). The samples in paraffin were sectioned at 2.5 *μ*m thickness, mounted onto glass slides (Carl Roth, Karlsruhe, Germany), and dried at 37°C overnight. First, sections with healthy and inflamed gingival tissues were stained with hematoxylin and eosin (H&E; Merck Eurolab, Darmstadt, Germany). Next, after deparaffinization and rehydration, selected sections were rinsed in PBS for 2 min. Subsequently, endogenous peroxidase was blocked using 0.3% methanol (AppliChem)/H_2_O_2_ (Merck Eurolab) solution for 5 min. Next, the sections were blocked with goat serum (Dako) for 20 min and incubated with rabbit primary polyclonal GHS-R antibody (Abcam; 1 : 100) in a humid chamber at 4°C overnight. Then, the sections were washed with PBS and incubated with goat anti-rabbit IgG-HRP secondary antibody (Dako) at RT for 30 min. The peroxidase activity was visualized with DAB chromogen (Thermo Fisher Scientific). Finally, all slides were rinsed with PBS and counterstained with Mayer's hematoxylin (Merck Eurolab) for 1 min. The images were collected using an Axioskop 2 microscope and analyzed with the AxioVision 4.7 software.

### 2.8. Statistical Analysis

The IBM SPSS Statistics software (Version 22, IBM SPSS, Chicago, IL, USA) was used for statistical analysis. Mean values and standard errors of the mean (SEM) were calculated for quantitative data. All experiments were performed in triplicate and repeated at least twice. For statistical comparison of the groups, the *t*-test, Mann–Whitney *U* test, and ANOVA followed by the post hoc Dunnett test were applied. Differences between groups were considered significant at *p* < 0.05.

## 3. Results

### 3.1. Regulation of GHS-R1a by *F. nucleatum* in Human PDL Cells

**Figure 1:**
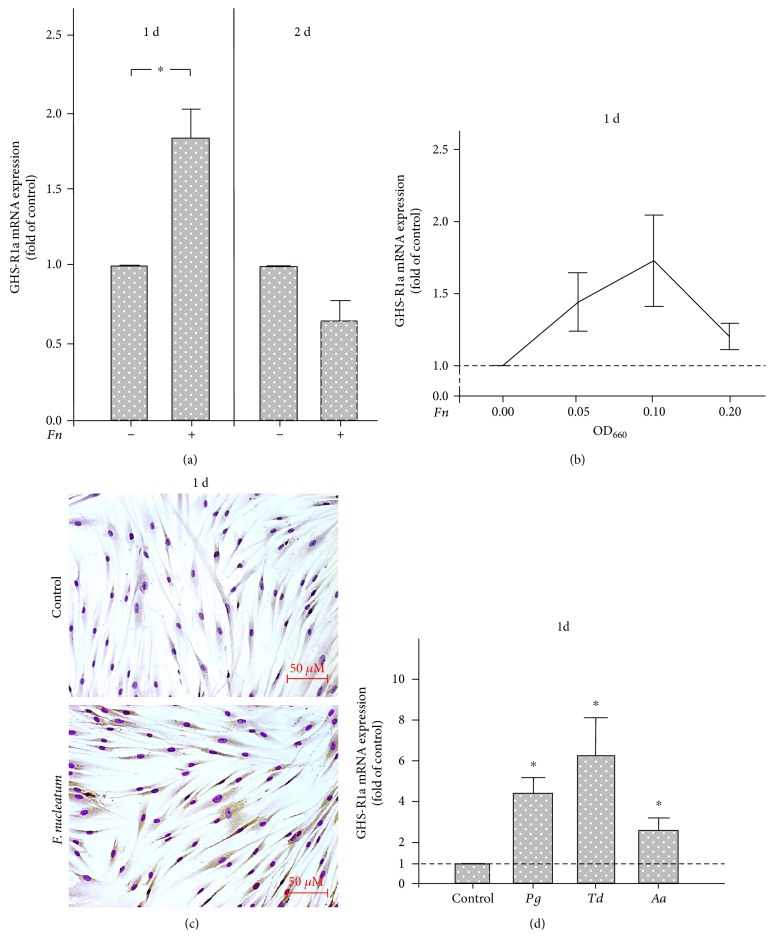
(a) Expression of GHS-R1a in the presence and absence of *F. nucleatum* (*Fn*; OD_660_: 0.1) in PDL cells at 1 d and 2 d, as analyzed by real-time PCR. Mean ± SEM (*n* = 9). ^∗^Significant (*p* < 0.05) difference between groups. (b) Expression of GHS-R1a in PDL cells exposed to various concentrations of *F. nucleatum* (*Fn*; OD_660_: 0.05, 0.1, and 0.2) at 1 d. Nonstimulated cells served as control. Mean ± SEM (*n* = 3). (c) GHS-R protein synthesis in PDL cells stimulated with *F. nucleatum* (OD_660_: 0.1) for 1 d, as visualized by immunocytochemistry. Nonstimulated cells served as control. Representative images from one out of three experiments are shown. (d) Expression of GHS-R1a in PDL cells exposed to *P. gingivalis* (*Pg*), *T. denticola* (*Td*), or *A. actinomycetemcomitans Y4* (*Aa*) (all OD_660_: 0.1) at 1 d, as determined by real-time PCR. Unstimulated cells served as control. Mean ± SEM (*n* = 24). ^∗^Significantly (*p* < 0.05) different from control.

First, we studied *in vitro* if GHS-R1a is expressed in PDL cells and, if so, whether this expression is regulated by the periodontopathogen *F. nucleatum*. Our experiments revealed that GHS-R1a was constitutively expressed in PDL cells and significantly upregulated by stimulation with *F. nucleatum* for 1 d. However, incubation of cells with *F. nucleatum* for a longer time, that is, 2 d, resulted in a remarkable downregulation of the receptor expression, as shown in Figure 1(a). Further experiments demonstrated that the short-term stimulatory effect of *F. nucleatum* on the GHS-R1a expression was dose-dependent, with the highest GHS-R1a expression levels at an OD_660_ of 0.1 at 1 d (Figure 1(b)). The stimulatory effect of *F. nucleatum* on GHS-R was also observed at protein level, as analyzed by immunocytochemistry. As depicted in Figure 1(c), higher GHS-R protein levels were found in *F. nucleatum-*stimulated cells as compared with the control. Notably, other periodontal pathogens were also capable of increasing the GHS-R1a expression in PDL cells. Incubation of cells with *P. gingivalis*, *T. denticola*, and *A. actinomycetemcomitans Y4* (OD_660_ = 0.1) increased significantly the GHS-R1a expression in PDL cells at 1 d (Figure 1(d)).

**Figure 2:**
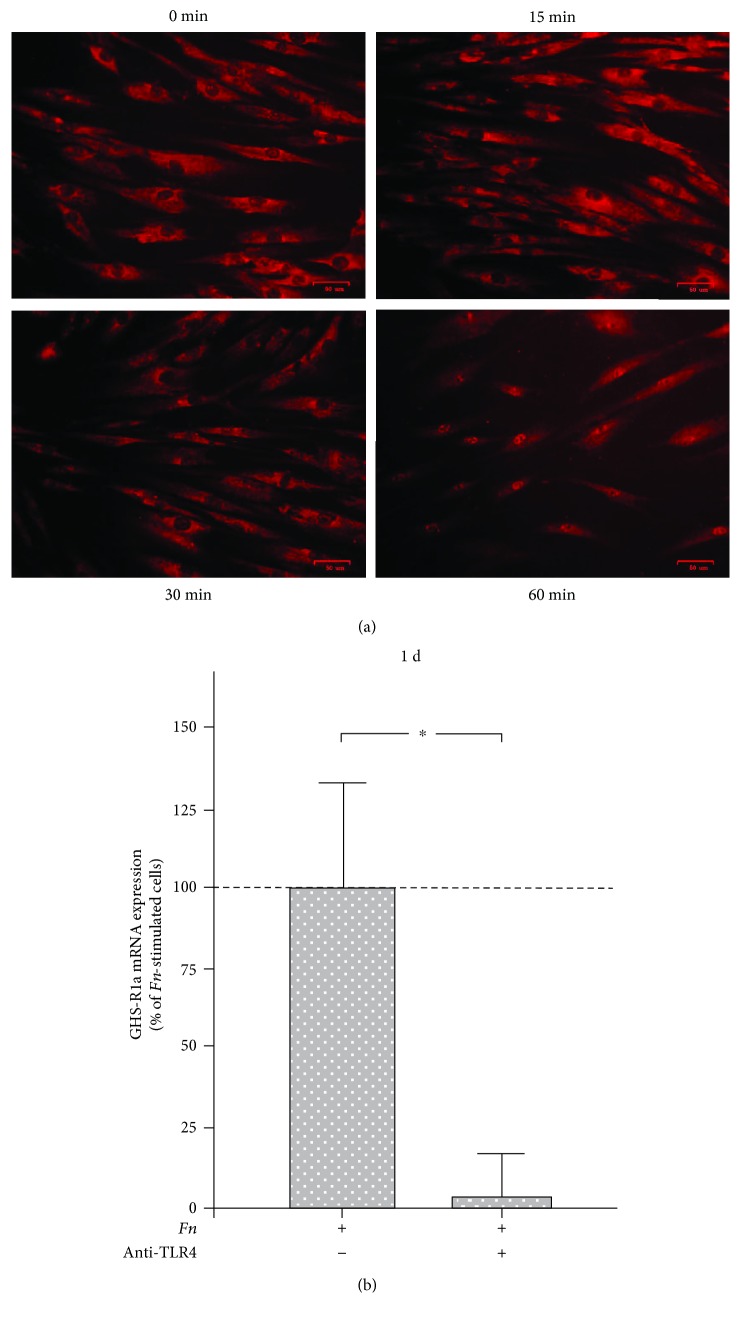
(a) Stimulation of NF-*κ*B (p65) nuclear translocation by *F. nucleatum* (OD_660_: 0.1) in PDL cells over 60 min, as analyzed by immunofluorescence microscopy. Representative images from one out of three experiments are shown. (b) Expression of GHS-R1a in PDL cells stimulated with *F. nucleatum* (*Fn*; OD_660_: 0.1) in the presence and absence of anti-TLR4 blocking antibody at 1 d, as determined by real-time PCR. Mean ± SEM (*n* = 3). ^∗^Significant (*p* < 0.05) difference between groups.

In another set of experiments, we sought to unravel the mechanisms underlying the stimulatory effect of *F. nucleatum* on GHS-R1a expression. As expected, *F. nucleatum* activated the NF-*κ*B signaling pathway and caused a maximal NF-*κ*B nuclear translocation at 60 min, as analyzed by immunofluorescence microscopy (Figure 2(a)). As our previous studies had demonstrated that *F. nucleatum* activates TLRs, which trigger the NF-*κ*B signaling pathway, we analyzed if the actions of *F. nucleatum* on GHS-R1a would also be dependent on TLRs [24]. When cells were preincubated with an anti-TLR4 blocking antibody, the stimulatory effect of *F. nucleatum* was almost completely abolished at 1 d, as shown in Figure 2(b).

### 3.2. Effects of GHRL on the Expressions of Cytokines and GHS-R1a in PDL Cells

**Figure 3:**
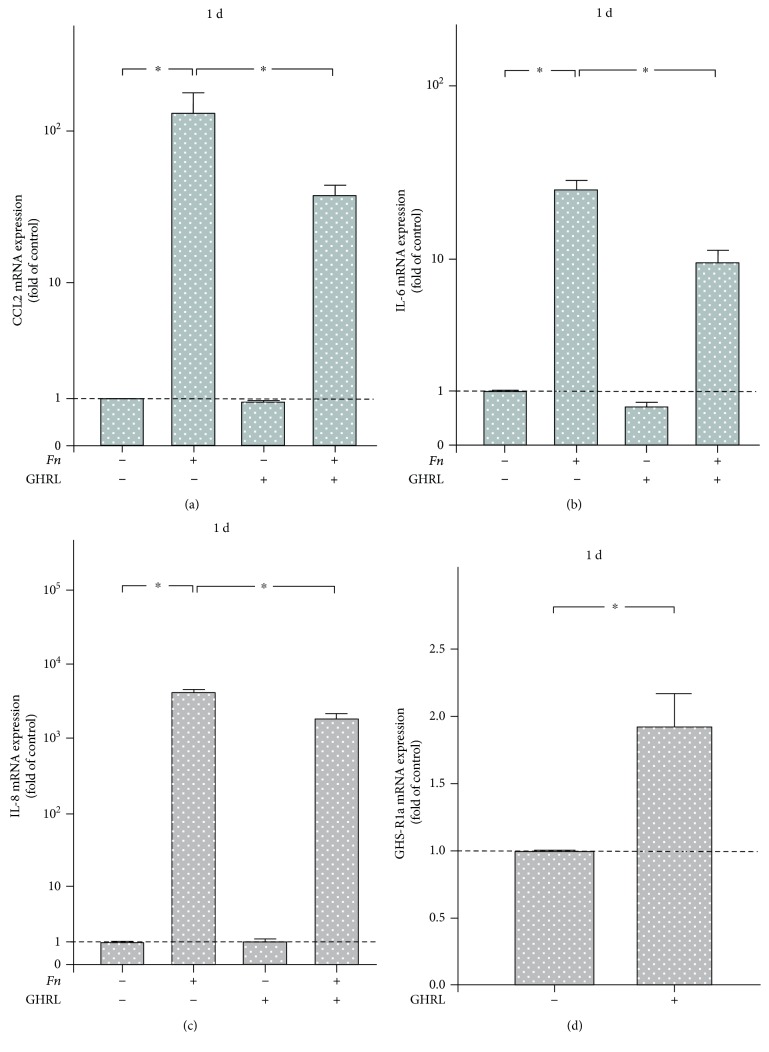
Expressions of CCL2 (a), IL-6 (b), and IL-8 (c) in PDL cells in the presence of *F. nucleatum* (*Fn*; OD_660_: 0.1) and/or GHRL (20 nM) at 1 d, as analyzed by real-time PCR. Unstimulated cells served as control. Mean ± SEM (*n* = 15). ^∗^Significant (*p* < 0.05) difference between groups. (d) Expression of GHS-R1a in PDL cells in the presence and absence of GHRL (20 nM) at 1 d. Mean ± SEM (*n* = 12). ^∗^Significant (*p* < 0.05) difference between groups.

Next, we sought to prove a possible anti-inflammatory nature of the GHRL/GHS-R system in PDL. Stimulation of PDL cells with *F. nucleatum* increased significantly the expressions of CCL2, IL-6, and IL-8 at 1 d, as expected. However, preincubation of the cells with GHRL counteracted significantly the stimulatory effects of *F. nucleatum* on the expressions of these proinflammatory/chemotactic cytokines, as depicted in Figures 3(a)–3(c). In the absence of *F. nucleatum*, no significant effects of GHRL on the cytokine expressions were observed (Figures 3(a)–3(c)). Interestingly, incubation of cells with GHRL increased significantly the expression of its own functional receptor at 1 d (Figure 3(d)).

### 3.3. Expression of GHS-R1a in Gingival Biopsies from Human and Rats

**Figure 4:**
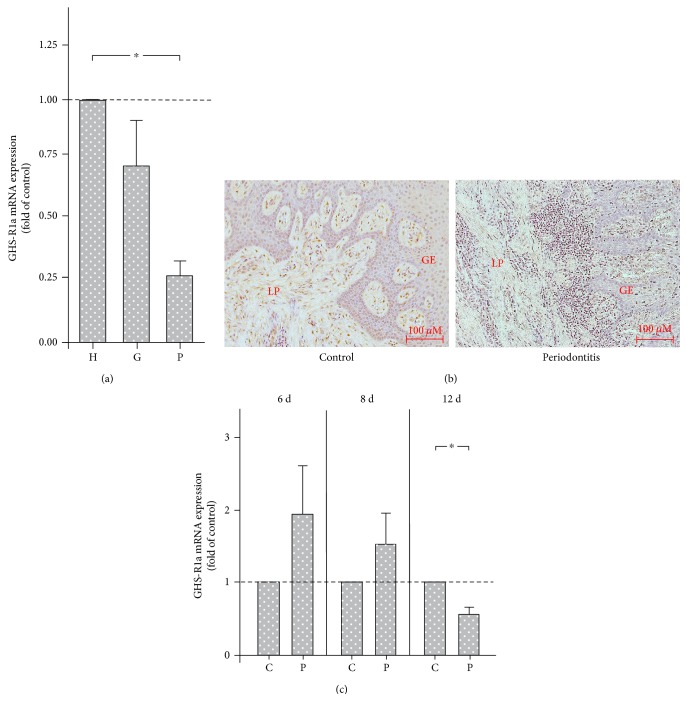
(a) GHS-R1a expression in human gingival biopsies from periodontally healthy (H), gingivitis (G), and periodontitis (P) sites, as analyzed by real-time PCR. Mean ± SEM (*n* = 10 donors/group). ^∗^Significant (*p* < 0.05) difference between groups. (b) GHS-R protein immunostaining in human gingival biopsies from periodontally healthy control and periodontitis sites, as visualized by immunohistochemistry. Representative images from one out of three donors of each group are shown. LP: lamina propria; GE: gingival epithelium. (c) GHS-R1a expression in rat gingival biopsies from periodontally healthy (C) and periodontitis (P) sites at 6 d, 8 d, and 12 d, as analyzed by real-time PCR. Mean ± SEM (*n* = 4 rats/group). ^∗^Significant (*p* < 0.05) difference between groups.

To study the expression and regulation of GHS-R1a under microbial conditions in a more complex environment, human gingival biopsies from periodontally healthy, gingivitis and periodontitis sites were collected and analyzed for the synthesis of GHS-R1a. As analyzed by real-time PCR, the GHS-R1a expression was significantly downregulated in gingival tissues from sites of periodontitis as compared with periodontally healthy sites. A reduced GHS-R1a expression was also found in gingival biopsies from gingivitis sites, even though the difference, as compared with periodontally healthy sites, did not reach significance (Figure 4(a)). The findings at transcriptional level were paralleled by observations at protein level. As revealed by immunohistochemistry, staining against GHS-R protein was more pronounced and frequently found in gingival biopsy samples collected from healthy sites, when compared with sites of periodontitis (Figure 4(b)). As GHS-R-positive cells, gingival fibroblasts of the lamina propria and gingival epithelial cells were identified.

Finally, we also sought to study the time course of GHS-R1a expression in a rat ligature-induced experimental periodontitis model. As shown in Figure 4(c), the GHS-R1a expression was higher at periodontitis sites than at healthy control sites at 6 d and 8 d, but the differences did not reach statistical significance. By contrast, gingival biopsies from sites of periodontitis showed significantly lower GHS-R1a levels, when compared with control sites, at 12 d (Figure 4(c)).

## 4. Discussion

Our *in vitro* and *in vivo* experiments provide original evidence that the expression of the functional ghrelin receptor in periodontal cells and tissues is modulated by periodontitis-associated microorganisms. Although GHS-R1a was initially upregulated, a continuous exposure of periodontal cells and tissues to periodontopathogens resulted in a GHS-R1a downregulation. Our experiments also demonstrated that GHRL inhibits the bacteria-induced upregulation of proinflammatory and chemotactic cytokines, thereby proving the anti-inflammatory nature of this peptide hormone. These findings therefore suggest that, due to the downregulation of the GHS-R1a by long-term exposure to periodontal bacteria, the anti-inflammatory actions of GHRL may be reduced in chronic periodontal infections, which could lead to an enhanced periodontal inflammation and destruction.

GHRL plays a critical role in a wide range of biological processes, such as the regulation of food intake, energy expenditure, body weight, sleep, and memory [12]. GHRL mediates its effects by binding to its functional receptor, that is, GHS-R1a [15]. Until now, very little is known about the physiology of the GHRL/GHS-R system in oral tissues. GHRL and its receptors have been detected in submandibular, parotid, and sublingual salivary glands as well as in oral epithelial cells and fibroblasts [21, 25, 26]. Moreover, GHRL can be found in saliva and GCF [17, 26]. Interestingly, GHRL levels in GCF from patients with chronic periodontitis were lower than those from healthy individuals. However, opposite findings were reported, when periodontally healthy and periodontitis subjects were also afflicted with type 2 diabetes [17]. Another study has shown elevated plasma levels of GHRL in patients with chronic periodontitis, as compared with periodontally healthy individuals [18]. The aforementioned studies point strongly at a potential role of the GHRL/GHS-R system in oral tissues.

Our *in vitro* experiments revealed that *F. nucleatum* upregulated the CCL2, IL-6, and IL-8 expressions. These cytokines exert proinflammatory and chemotactic effects and have been shown to be increased at sites of periodontitis as compared with periodontally healthy sites [27, 28]. Our findings concur with observations by other investigators who have shown increased cytokine productions by neutrophil-like cells and macrophages in response to *F. nucleatum* [29, 30]. Notably, GHRL inhibited the *F. nucleatum*-induced upregulation of these cytokines in periodontal cells, thereby proving the anti-inflammatory nature of this peptide hormone. The finding that GHRL can cause a downregulation of stimulated cytokine expressions has also been observed in other cells and species. For example, it has been shown that GHRL counteracts the stimulatory effects of lipopolysaccharide (LPS) on the IL-6 and IL-8 releases from mouse dopaminergic neurons and human oral epithelial cells, respectively [26, 31]. Similarly, GHRL inhibited the endotoxin-induced IL-8, TNF*α*, and CCL2 syntheses in rats [32]. In addition, GHRL abolished the stimulatory actions of TNF*α* and angiotensin II on the cytokine production by human umbilical vein endothelial cells [32, 33]. Interestingly, the anti-inflammatory actions of GHRL seem to involve the NF-*κ*B pathway [33, 34]. The studies mentioned above support our findings which suggest that GHRL may play an important role in controlling periodontal inflammation.

In the present *in vitro* study, *F. nucleatum* was used to mimic an infectious environment, as in our previous experiments [24, 35]. *F. nucleatum* represents a gram-negative, anaerobic microorganism which functions as a bridge bacterium between early and late colonizers during biofilm development. It is associated with both gingivitis and periodontitis, invades periodontal cells, and supports other periodontal bacteria to invade host cells [36–38]. Periodontitis is a mixed polymicrobial disease, and other bacterial species are involved. Interestingly, the periodontitis-associated bacteria *P. gingivalis*, *T. denticola*, and *A. actinomycetemcomitans* also caused an upregulation of the GHS-R1a. Nevertheless, further studies are necessary to analyze the effects of a mixed bacterial biofilm on the GHRL/GHS-R system. Moreover, the bacteria used in our experiments were lysed and, therefore, nonvital. Although LPS might have been a major compound of this lysate, other virulence factors may also have contributed to the stimulatory effects of the bacteria used in our experiments, which should be investigated in further studies.

Since the Toll-like receptor- (TLR-) NF-*κ*B pathway is involved in the expression of proinflammatory cytokines, we also studied the effects of *F. nucleatum* on this signaling pathway in PDL cells. *F. nucleatum* stimulated the nuclear translocation of NF-*κ*B in PDL cells, which is consistent with previous reports [24]. Studies in neutrophil-like cells, colorectal cancer cells, and macrophages have also shown that *F. nucleatum* exploits this pathway for its effects on the production of cytokines, thereby supporting our observation [29, 30, 39]. Since the NF-*κ*B pathway can be activated by TLR4, we next preincubated PDL cells with an anti-TLR4 blocking antibody. Our experiments showed that the initial stimulatory effect of *F. nucleatum* on the GHS-R1a expression was indeed dependent on TLR4. Whether additional pathways are involved in the actions of *F. nucleatum* on the GHRL/GHS-R system should be unraveled in future studies.

To investigate the GHS-R1a expression in periodontal cells in a more complex environment, gingival biopsies from periodontitis patients and periodontally healthy individuals were analyzed [22]. The weakest GHS-R1a expression was found in biopsies from subjects with periodontitis. The GHS-R1a expression in gingival tissues from gingivitis subjects was also lower than that from periodontally healthy subjects, but higher than that from periodontitis patients. These findings show clearly a dose-dependent GHS-R1a downregulation in gingival tissues. Furthermore, as analyzed by immunohistochemistry, the GHS-R protein was also reduced in gingiva from periodontitis patients, which paralleled our findings at transcriptional level. Moreover, the histological analyses revealed that the GHS-R1 is not only produced by fibroblasts but also by epithelial cells. To monitor the GHS-R1a expression in periodontal cells and tissues under bacterial condition over time, a rat ligature-induced periodontitis model was applied. The ligature-induced plaque accumulation led to a significant periodontal inflammation and tissue destruction [23]. In this *in vivo* model, the GHS-R1a expression in gingival samples was initially upregulated, which is in accordance with our *in vitro* data. However, chronic exposure of the periodontal tissues to the ligature-induced plaque accumulation caused a significant downregulation of GHS-R1a, confirming our findings from human gingival biopsies. These *in vivo* data confirm and expand our *in vitro* results, demonstrating that a long-term incubation of periodontal cells and tissues with periodontal bacteria causes a downregulation of the functional receptor for the anti-inflammatory peptide GHRL.

In the *in vitro* experiments, PDL cells were used, as they play a critical role in periodontal destruction and regeneration. The PDL cells had been phenotyped prior to their use to confirm their ability to differentiate in osteoblastic cells. Since no osteogenic medium was used in the present *in vitro* experiments, these cells attained a fibroblastic phenotype, which facilitated the comparison with the gingival tissue samples from human and rats.

## 5. Conclusions

Our study provides novel evidence that the GHS-R1a expression in periodontal cells is modulated by periodontitis-associated microorganisms. Despite an initial GHS-R1a upregulation, which may serve as a protective cellular response, a continuous exposure of periodontal cells to periodontopathogens results in a GHS-R1a downregulation. Our experiments also demonstrated that GHRL inhibits the bacteria-induced expression of proinflammatory cytokines in periodontal cells. Therefore, due to the downregulation of the functional ghrelin receptor, the anti-inflammatory actions of GHRL may be diminished in chronic periodontal infections, which could lead to an enhanced periodontal inflammation and tissue destruction.
